# Validation of qPCR Methods for the Detection of *Mycobacterium* in New World Animal Reservoirs

**DOI:** 10.1371/journal.pntd.0004198

**Published:** 2015-11-16

**Authors:** Genevieve Housman, Joanna Malukiewicz, Vanner Boere, Adriana D. Grativol, Luiz Cezar M. Pereira, Ita de Oliveira e Silva, Carlos R. Ruiz-Miranda, Richard Truman, Anne C. Stone

**Affiliations:** 1 School of Human Evolution and Social Change, Arizona State University, Tempe, Arizona, United States of America; 2 Departamento de Bioquímica e Biologia Molecular, Universidade Federal de Viçosa, Viçosa, Minas Gerais, Brazil; 3 Laboratório de Ciências Ambientias, Centro de Biociências e Biotecnologia, Universidade Estadual do Norte Fluminense, Campos dos Goytacazes, Rio de Janeiro, Brazil; 4 Centro de Conservação e Manejo de Fauna da Caatinga, Universidade Federal do Vale do São Francisco, Petrolina, Pernambuco, Brazil; 5 Departamento de Biologia Animal, Universidade Federal de Viçosa, Viçosa, Minas Gerais, Brazil; 6 HHS\HRSA\HSB National Hansen's Disease Program-NIAID IAA-2646, Baton Rouge, Louisiana, United States of America; University of Tennessee, UNITED STATES

## Abstract

Zoonotic pathogens that cause leprosy (*Mycobacterium leprae*) and tuberculosis (*Mycobacterium tuberculosis* complex, MTBC) continue to impact modern human populations. Therefore, methods able to survey mycobacterial infection in potential animal hosts are necessary for proper evaluation of human exposure threats. Here we tested for mycobacterial-specific single- and multi-copy loci using qPCR. In a trial study in which armadillos were artificially infected with *M*. *leprae*, these techniques were specific and sensitive to pathogen detection, while more traditional ELISAs were only specific. These assays were then employed in a case study to detect *M*. *leprae* as well as MTBC in wild marmosets. All marmosets were negative for *M*. *leprae* DNA, but 14 were positive for the mycobacterial rpoB gene assay. Targeted capture and sequencing of rpoB and other MTBC genes validated the presence of mycobacterial DNA in these samples and revealed that qPCR is useful for identifying mycobacterial-infected animal hosts.

## Author Summary

Mycobacterial pathogens that cause tuberculosis and leprosy can be detected in wild animal populations using non-invasive cheek swab samples and quantitative polymerase chain reaction assays that target specific portions of mycobacterial genomes. A preliminary study in armadillos confirms that using multiple assays in tandem is optimal for surveying infection in animals, and a case study identifies some mycobacterial infection in wild marmosets from Brazil. These validated methods can be used to advance our understanding of the distribution and impact of zoonotic pathogens on wild animal populations.

## Introduction

Through the two-way transmission of pathogens between animals and humans, zoonotic diseases have a tremendous impact on modern human populations [1–2]. *Mycobacterium* is one important group of bacteria that has a long history of zoonosis [3]. In particular, the *Mycobacterium tuberculosis* complex (MTBC) and *Mycobacterium leprae* respectively cause tuberculosis and leprosy—diseases that have afflicted humans for centuries and continue to contribute to major public health issues today [4]. Additionally, some mycobacterial pathogens can be transmitted among other animals, so it is important to identify the presence of these pathogens in potential animal reservoirs to assess the potential for disease spread and exposure threats.

The progressive respiratory disease tuberculosis is caused by several MTBC species, including *M*. *tuberculosis*, *M*. *canettii*, *M*. *bovis*, *M*. *africanum*, and *M*.*microti* [5] and can infect ungulates, carnivores, rodents, bats, marsupials, and primates [5–8]. Within the primate order, apes, baboons, rhesus macaques, colobus monkeys, mangabeys, langurs, spider monkeys, wooly monkeys, and capuchins can all harbor *M*. *tuberculosis* and display tuberculosis symptoms [5,8]. Similarly, the chronic disease leprosy, which primarily affects the skin, peripheral nerves, and upper airways, can also infect armadillos, chimpanzees, cynomolgus macaques, sooty mangabeys, rhesus macaques, and African green monkeys [9–12]. The abundance of non-human primate mycobacterial reservoirs is likely due to the relatively weak interspecific barrier to zoonotic disease transfer between human and non-human primates [2]. In order to assess human disease spread and exposure threats, it is important to survey the spread of zoonotic mycobacterial infections across primate populations, especially those with close physical proximity to humans [1,13].

However, diagnosing such mycobacterial infections in animals is difficult. Many animal tissue collection protocols are invasive and hazardous to investigators, and techniques for detecting the presence of mycobacteria vary in their usefulness and effectiveness. Sample cultures require long incubation times, species-specific serological tests do not exist, and cross-reactions or a lack of specificity lead to false-positive and false-negative results [14]. Despite these complications, several methods have been developed to detect the presence of mycobacteria in animal reservoirs. Initially, skin tests involving intradermal injection of tuberculin were performed [7], but generally gave unreliable results. Serological assays to detect mycobacteria specific antibodies and other immunological markers such as enzyme-linked immunosorbent assay (ELISA) [7,13–14] were then standardized and continue to be used today as reliable methods. Finally polymerase chain reaction (PCR) assays and sequencing techniques [5,13–15] were designed to identify species-specific mycobacterial DNA in zoonotic animal reservoirs.

Optimal surveys of mycobacterial infection in wild animal populations should include non-invasive sample collection protocols in combination with fast and relatively cost effective assays that maximizes detection sensitivity and specificity, or the proportion of infected animals accurately identified as infected and the proportion of non-infected animals identified as not infected, respectively. This study tests and employs such a technique which combines cheek swab collection with quantitative polymerase chain reaction (qPCR) assays for detecting single- and multi-copy loci specific to mycobacterial species. The targeted loci were described in previous studies [4,15–18] but have been combined for the first time in this study. Specifically, the efficiency of this single- and multi-copy loci qPCR method for detecting *M*. *leprae* in experimentally infected and non-infected armadillos is compared to that of more traditional ELISAs. Once validated, this qPCR method is used to assess both *M*. *leprae* and MTBC infection in wild marmosets [19].

Armadillos were chosen for this study because they are known natural hosts of *M*. *leprae*, and zoonotic disease transfers have resulted from their close physical proximity with humans. Marmoset ranges overlap with those of armadillos, and therefore, marmosets, especially those living in close proximity to humans, may also serve as potential mycobacteria zoonotic reservoirs. Bacterial infection is a primary cause of mortality in marmosets [20], but the impact of mycobacterial disease in these primates has not been examined yet. This study suggests that marmosets can harbor mycobacteria. Target capture and next-generation sequencing further support these findings and indicate that a combined single- and multi-copy loci qPCR method from cheek swab samples is a rapid and dependable screening tool for mycobacterial infections in wild animal populations.

## Methods

### Trial Study: Validation of Mycobacterial qPCR Methods

#### Samples

A total of 25 armadillos from the National Hansen’s Disease (Leprosy) Research Program were experimentally infected with *M*. *leprae* using the protocol outlined in [21], while 20 remained non-infected (Table 1). Experimental infection was monitored using serology, and animals were diagnosed as successfully infected after 6–9 months when levels of IgM antibodies to PGL1 (Bioresources, Manassas, VA) reached an O.D. cut off value of 0.700 and levels of IgG antibodies to LID1 (IDRI, Seattle, WA) reached a. O.D. cut off of 0.300. Only successfully infected armadillos were included in the experimentally infected cohort (n = 25). Further validation through cell culture or AFB staining was not performed, since leprosy bacteria are unable to be grown in culture and the infection status of each animal was known. For both infected and non-infected armadillos (n = 45), blood and cheek swab samples for ELISAs and qPCRs, respectively, were collected while anesthetized.

**Table 1:** Study sample sets.

**Armadillo Samples**	**Number**
Experimentally Infected with *M*. *leprae*	25
Not Experimentally Infected with *M*. *leprae*	20
**Marmoset Samples**	**Number**
*Callithrix jacchus*	10
Recife, Pernambuco (n = 10)	
Hybrid	49
Petrolina, Pernambuco / Juazeiro, Bahia (n = 20)	
Silva Jardim and Rio Bonito, Rio de Janeiro (n = 29)	
*Callithrix penicillata*	26
Brasília, Federal District (n = 15)	
Muriaé, Minas Gerais (n = 3)	
Recife, Pernambuco (n = 2)	
Goiania, Goiás (n = 6)	
*Callithrix* sp.	13
Brasília, Federal District (n = 1)	
Ipatinga, Minas Gerais (n = 12)	

Sample sets used in trial and case studies. Armadillo blood and cheek swab samples came from the National Hansen’s Disease (Leprosy) Research Program and were experimentally infected or not infected with *M*. *leprae*. Marmoset cheek swab samples came from wild populations in various regions of Brazil.

#### Ethics statement

Armadillos were housed at the National Hansen's Disease Program (NHDP) laboratory under a protocol approved by the NHDP Institutional Animal Care and Use Committee (Assurance A3032-01) in accordance with PHS policy for humane care and use of laboratory animals as set forth in the 8th edition of the PHS Guide.

#### Methods

DNA from cheek swab samples of both experimentally infected and non-infected armadillos was isolated using a phenol-chloroform extraction protocol [22]. The presence of *M*. *leprae* infection in each armadillo blood extract was examined with two separate ELISAs, one targeting the IgM antibody to the *M*. *leprae*-specific PGL1 antigen [23] and one targeting the IgG antibody to the *M*. *leprae*-specific LID1 antigen [24]. The presence and quantity of *M*. *leprae* DNA in each armadillo cheek swab extract were examined with two separate TaqMan qPCR assays, one targeting the 85B single-copy *M*. *leprae* gene [16] and one targeting the rlep multi-copy *M*. *leprae* gene [15] (Tables A and B in S1 Text). The fluorogenic 5' nuclease chemistry and 3’ minor groove binding ability of the MGB TaqMan probes allowed for specific targeting and amplification of the small gene fragments of interest. DNA extracts were tested for each targeted locus by comparison to non-template controls and a standard curve of serial dilutions created from genomic DNA of *M*. *leprae* TN in a 10-fold serial dilution of 1 to 100,000 copy numbers per microliter, optimized for each assay. Depending on availability, two or more replicates of each unknown sample, standard, and non-template control were included in each qPCR run. Reactions were in a 20μL total volume; 10μL of TaqMan 2x Universal Master Mix, primers, probe, 10mg/mL RSA, and 2μL of sample. Applied Biosystems 7900 thermocycling conditions included an initial hold at 50°C for 2 minutes, followed by enzyme activation at 95°C for 10 minutes and 40–50 cycles of amplification at 95°C for 15 seconds and 60°C for 1 minute. A qPCR inhibition test was also performed in a subset of armadillos to ensure that no major inhibitory effects were observed in samples (Table C in S1 Text).

#### Analysis

All analyses were performed blind and then compared to known infectious statuses. PGL1 and LID ELISAs were positive if antibody levels were greater than O.D. 0.700 and O.D. 0.300 respectively. qPCR results were analyzed with SDS 2.3. qPCR efficiency, which indicates how well the slope of the standard curves match an ideal slope, was calculated as efficiency = -1+10(-1/slope) to determine if the assay fell within an acceptable range of 90–100%. Replicates were identified as positive if the amplification plot curves had appropriate exponential, linear, and plateau phases that crossed a manually designated threshold and if the multicomponent plot reporter dye readings increased over time. Infection status was determined using three independent cut-off levels: greater than 75% positive replicates for the 85B qPCRs, greater than 75% positive replicates for the rlep qPCRs, and greater than 50% positive runs for 85B and rlep qPCRs in combination indicated positive *M*. *leprae* detection. Chi-square goodness of fit tests were performed to determine how well each test correctly identified infected armadillos. Lastly, the sensitivity (proportion of infected armadillos accurately identified as infected), specificity (proportion of non-infected armadillos identified as not infected), false positive rates (FPR, proportion of non-infected armadillos inaccurately identified as infected), and false negative rates (FNR, proportion of infected armadillos inaccurately identified as not infected) for the ELISAs and qPCRs were assessed.

### Case Study: Survey of Mycobacteria in Wild Marmosets

#### Samples

Cheek swab samples were collected from anesthetized wild marmosets—classified as *Callithrix jacchus*, *Callithrix penicillata*, or a hybrid of these two species based on phenotypic and genetic data [25]. Individuals unable to be grouped into one of these taxa were classified as *Callithrix* sp. Marmosets (n = 98) were found in several locations across Brazil–(1) Goiania, Goiás; (2) Brasília, Federal District; (3) the neighboring municipalities of Petrolina, Pernambuco and Juazaiero, Bahia; (4) Recife, Pernambuco; (5) Ipatinga, Minas Gerais; (6) Muriaé, Minas Gerais; and (7) the neighboring cities of Silva Jardim and Rio Bonito in Rio de Janeiro. Samples were grouped according to species and location (Table 1), and sampling was performed as discussed in [25].

#### Ethics statement

All marmoset cheek swab sample collection, exportation, and importation were approved by ASU IACUC (protocol #11-1150R), the Brazilian Ministry for the Environment and Natural Resources (IBAMA, protocol #28075–2), and by a CITES export permit (protocol #11BR007015/DF).

#### Methods

DNA from cheek swabs was isolated using a phenol-chloroform extraction protocol [22]. The presence and quantity of *M*. *leprae* DNA in each marmoset cheek swab extract were examined using the same techniques as described above. The presence and quantity of MTBC DNA in each extract were examined with three separate TaqMan qPCR assays, two targeting the single-copy rpoB gene—rpoB1 and rpoB2 [17]–and one targeting the MTBC-specific multi-copy IS6110 insertion element [4,18] (Tables A and B in S1 Text). The rpoB1 primer/probe set can amplify species within the MTBC and some species outside of this complex [17], making it good for identifying general mycobacterial species, but not specific strains. Conversely, rpoB2 is specific to the MTBC [17]. Extracts were tested for all MTBC targeted loci by comparison to a standard curve of serial dilutions created from genomic DNA of *M*. *tuberculosis* H37Rv in a 10-fold serial dilution of 1 to 100,000 copy numbers per microliter, optimized for each assay. All other MTBC qPCR methods were similar to those defined for the *M*. *leprae* qPCRs. A qPCR inhibition test was also performed in a subset of marmosets to ensure that no major inhibitory effects were observed in samples (Table C in S1 Text).

#### Analysis

qPCR results were analyzed with SDS 2.3, and qPCR efficiency was calculated as described above. Both amplification and multicomponent plots were used to classify sample replicates as positive or negative. Lastly, marmosets were classified as positive for mycobacterial infection if greater than 60% of the replicates over all runs were positive based on both the amplification and multicomponent plots and negative for mycobacterial infection if less than 60% of the replicates over all runs were positive based on either the amplification or multicomponent plots.

#### Sequencing

Marmosets classified as harboring mycobacterial DNA and containing the highest initial mycobacterial genome copy numbers (n = 8) were further analyzed using next-generation sequencing techniques. DNA libraries were prepared according to [26–27] and enriched as in [28] for MTBC DNA using baits designed to capture the MTBC rpoB, katG, gyrA, gyrB, and mtp40 genes and sequenced on an Illumina MiSeq Nano. Reads (S1 Dataset) were merged and quality filtered following [27], and resulting sequences were aligned to mycobacterial reference sequences using BWA [29] and further analyzed using the metagenomics program MEGAN with default settings [30].

## Results

### Trial Study: Validation of Mycobacterial qPCR Methods

**Fig 1:**
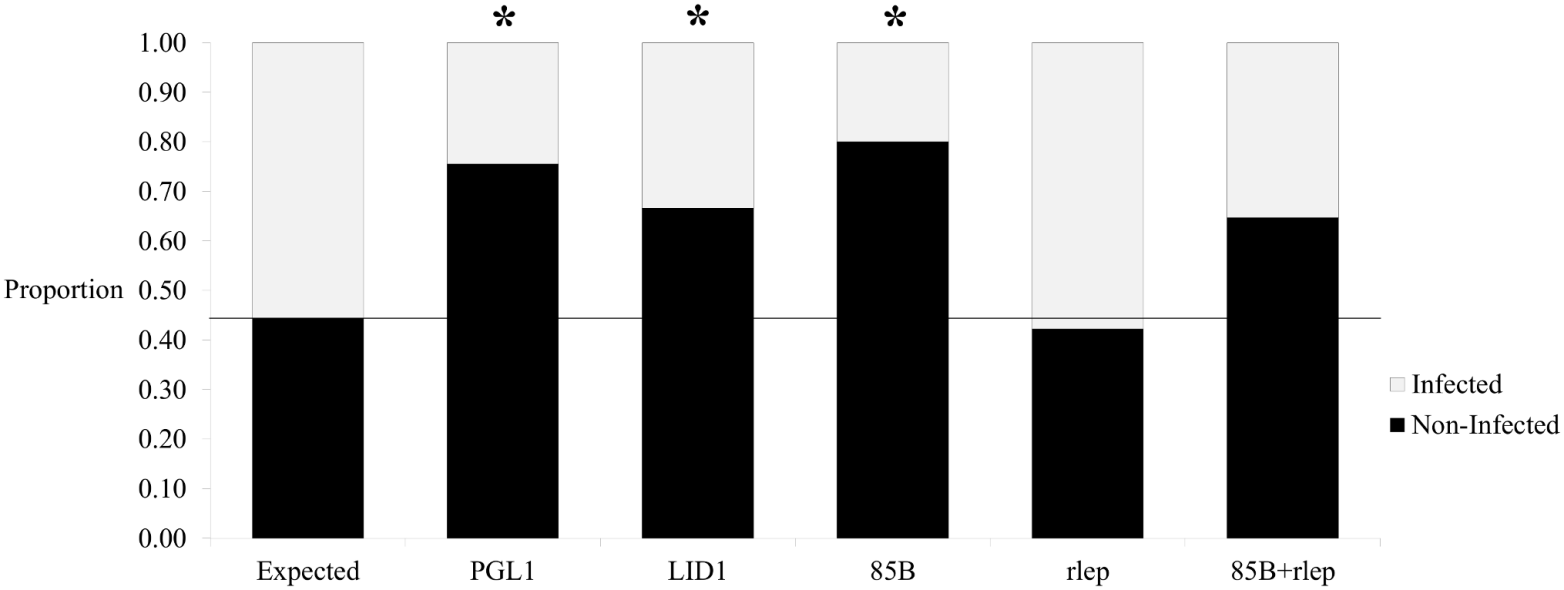
Goodness of fit analysis for trial study. ELISA and qPCR test results for the armadillo trial study as compared to the expected results based on known armadillo experimental infection statuses (infected n = 25, non-infected n = 20). Each assay type is compared to the expected using Chi-squared analyses. Assay types include the PGL1 ELISA (infected n = 11, non-infected n = 34, χ2 = 17.52, p<0.01), LID1 ELISA (infected n = 15, non-infected n = 30, χ2 = 8.92, p<0.01), 85B qPCR (infected n = 9, non-infected n = 36, χ2 = 22.90, p<0.01), rlep qPCR (infected n = 26, non-infected n = 19, χ2 = 0.12, p = 0.73), and combination of 85B and rlep qPCRs (85B+rlep, infected n = 18, non-infected n = 27, χ2 = 4.36, p = 0.04). Chi-squared analyses show all tests significantly differ from the expected (*p<0.01) except the qPCR tests performed using only the rlep target and the combination of 85B and rlep.

**Fig 2:**
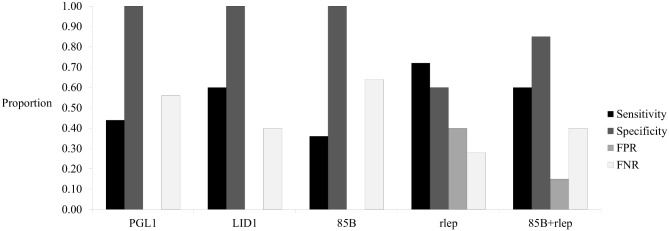
Sensitivity, specificity, false positive rate, and false negative rate determined in trial study. Analysis of the sensitivity (proportion of infected armadillos accurately identified as infected), specificity (proportion of non-infected armadillos identified as not infected), false positive rates (FPR, proportion of non-infected armadillos inaccurately identified as infected), and false negative rates (FNR, proportion of infected armadillos inaccurately identified as not infected) of ELISAs using blood samples and qPCRs using cheek swab samples. Assay types include the PGL1 ELISA (sensitivity = 0.44, specificity = 1.00, FPR = 0.00, FNR = 0.56), LID1 ELISA (sensitivity = 0.60, specificity = 1.00, FPR = 0.00, FNR = 0.40), 85B qPCR (sensitivity = 0.36, specificity = 1.00, FPR = 0.00, FNR = 0.60), rlep qPCR (sensitivity = 0.72, specificity = 0.60, FPR = 0.40, FNR = 0.28), and combination of 85B and rlep qPCRs (85B+rlep, sensitivity = 0.60, specificity = 0.85, FPR = 0.15, FNR = 0.40).

When compared to the known number of armadillos positively infected with *M*. *leprae*, all tests significantly differ from expected (p<0.01) except for the qPCR targeting only the multi-copy rlep gene (χ2 = 0.12, p = 0.73) and the combination of this qPCR with the single-copy 85B gene (χ2 = 4.36, p = 0.04) (Fig 1). ELISAs for the PGL1 and LID1 antigens are extremely specific but not highly sensitive with moderate FNRs (Fig 2). Conversely, qPCR results are specific and sensitive for the single-copy 85B gene and multi-copy rlep gene, respectively, and also have moderate FNRs and FPRs, respectively (Fig 2). These results suggest that unlike ELISAs, qPCRs using cheek swab samples are able to be both highly sensitive and specific in population infection surveys.

### Case Study: Survey of Mycobacteria in Wild Marmosets

**Fig 3:**
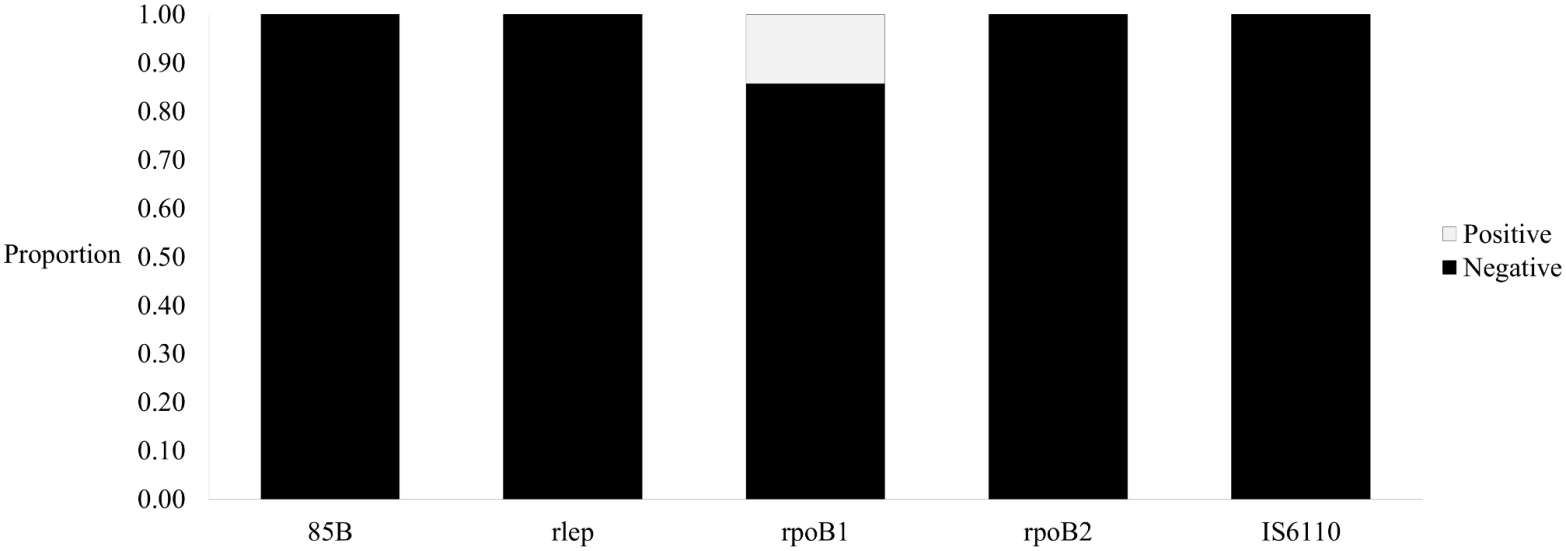
Overall qPCR results for case study. qPCR results for marmoset samples (n = 98). All marmosets were negative for the *M*. *leprae*-specific 85B and rlep genes and the MTBC-specific rpoB2 and IS6110 genes. However, 14 are positive for the mycobacterial rpoB1 gene. Approximately 0.001–0.137 mycobacterial genome copies per ng of DNA extracted were identified from these rpoB1-positive samples.

**Fig 4:**
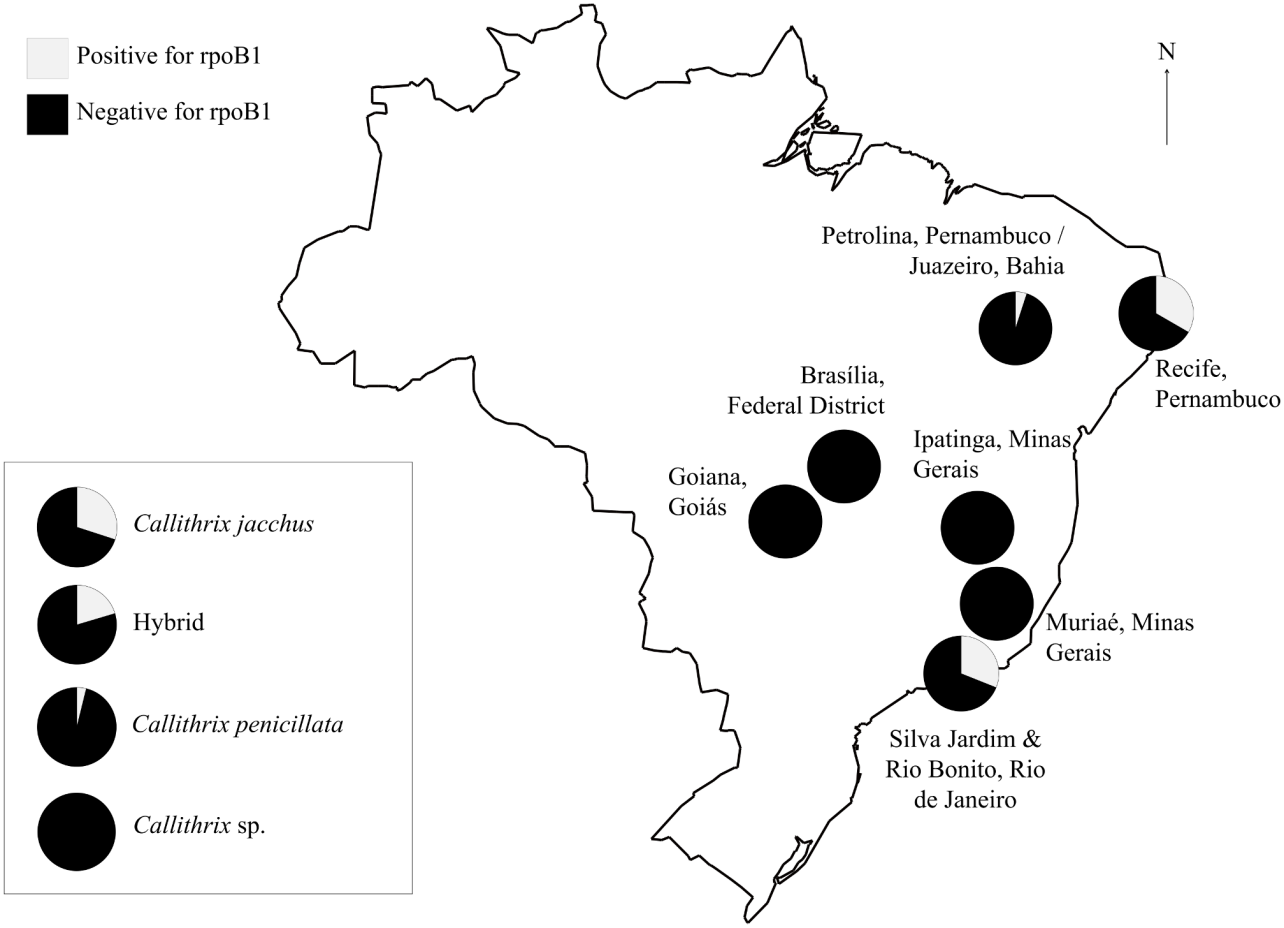
Distribution of mycobacteria identified in case study. Geographic and taxonomic distribution of rpoB1 positive marmoset samples. rpoB1 positive samples are found in Recife, Pernambuco (n = 4), the neighboring municipalities of Petrolina, Pernambuco and Juazeiro, Bahia (n = 1), and the neighboring cities of Silva Jardim and Rio Bonito, Rio de Janeiro (n = 9) and in *C*. *jacchus* (n = 3), hybrids (n = 10), and *C*. *penicillata* (n = 1).

All 98 tested marmosets were negative for 85B, rlep, rpoB2, and IS6110. However, 14 were positive for the rpoB1 locus (Fig 3) with 0.001–0.137 mycobacterial copies per nanogram of DNA extracted, based on the *M*. *tuberculosis* genome size of 4,410,000bp. Marmosets potentially harboring mycobacteria were found in the neighboring cities of Silva Jardim and Rio Bonito in Rio de Janeiro, the neighboring municipalities of Petrolina, Pernambuco and Juazaiero, Bahia, and at an animal triage center in Recife, Pernambuco, and included *C*. *jacchus*, *C*. *penicillata*, and hybrids of those species (Fig 4). General sequence results were averaged across all marmoset samples (n = 8) and a blank control (n = 1) (Table 2). Five genes were enriched before sequencing, and the estimated reads per gene takes this into consideration. BWA and MEGAN results were somewhat inconclusive but revealed that no sample reads aligned to *M*. *tuberculosis* while some could be assigned to other species within the genus *Mycobacterium* (Table 3). Sequence reads were also found in the blank sample. However, these were significantly shorter than the sample reads and did not align to any mycobacterial sequences. A small portion of reads (n = 24) aligned to Actinobacteria, Firmicutes, Proteobacteria, and Eukaryota. Nevertheless, the majority of reads (n = 119) had no hits in MEGAN and were likely primer and adapter dimers.

**Table 2:** Summary of sequencing results.

	Marmoset Samples	Blank Sample
**Average Number of Reads (minimum—maximum)**	5,796 (1,526–14,598)	154
**Average Estimated Number of Reads per Gene (minimum—maximum)**	1,159 (305–2,920)	31
**Average Range of Sequence Lengths**	12bp–288bp	5bp–163bp

Overview of sequence read quantity and quality from targeted capture and sequencing of samples. Marmoset samples are n = 8, and blank sample is n = 1.

**Table 3:** Summary of MEGAN results.

**Pathogenic Species**	**Marmoset Samples**	**Blank Sample**
*M*. *avium*	88	0
*M*. *leprae*	1	0
**Non-Pathogenic Species**	**Marmoset Samples**	**Blank Sample**
*M*. *gilvum*	1	0
*M*. *chubuense*	1	0
*M*. *neoaurum*	1	0
*M*. *smegmatis*	1	0
*M*. *vanbaalenii*	3	0
*M*. *sp*.*FI-13364*	1	0
*M*. *sp*. *JDM601*	5	0
*M*. *sp*. *VKM Ac-1817D*	1	0
**Genus**	**Marmoset Samples**	**Blank Sample**
Mycobacterium	113	0

Maximum number of sequence reads assigned to specific mycobacteria using MEGAN out of the total number of reads possible, as described in Table 2. Marmoset samples are n = 8, and blank sample is n = 1. The presence of the genus *Mycobacterium* is somewhat supported, while the presence of individual mycobacterial species is not well supported.

## Discussion

In the New World, *M*. *leprae* naturally transfers between humans and armadillos, and this study takes advantage of this known zoonotic pathway to identify a useful method for surveying mycobacterial infection in wild primate populations. This paper both validated this single- and multi-copy loci qPCR method for the detection of mycobacterial pathogens in animal hosts using non-invasive cheek swab samples and further tested this technique in a case study to assess mycobacterial infections in wild marmosets.

The trial study reveals that ELISAs are extremely specific but not highly sensitive, while qPCRs are both specific and sensitive for the single-copy 85B and multi-copy rlep genes, respectively (Figs 1 and 2). This suggests that a combination of single- and multi-copy loci qPCRs may be more effective at surveying animal populations for potential mycobacterial infection than ELISAs or other currently available techniques. Taqman qPCR technology is also a reliable method as it uses a non-extendable DNA probe which hybridizes to a region within the amplicon and reduces the amplification of non-specific molecules [31]. However, proper analysis of qPCR data depends on user-defined thresholds and baselines, so this method is subject to human errors. Therefore, qPCR is best used for target sequence presence/absence determination rather than copy number quantification, making it an extremely useful screening tool to be followed up by more rigorous methods for subsequent downstream analyses.

In the case study, all 98 marmosets were negative for *M*. *leprae* DNA. These marmosets were not necessarily expected to harbor *M*. *leprae*, but since leprosy is a prevalent disease in the New World [32–34], zoonotic transmission of this disease to marmosets is a possibility. In particular, Brazil has the highest prevalence of leprosy among humans in the Americas [35]. Leprosy is also found in New World armadillos, which serve as predominant natural hosts for this disease [36]. Since armadillo ranges extend over most of South and Central America and parts of North America [36] and since zoonotic transfers of *M*. *leprae* between armadillos and humans have been documented [9], contact between marmosets and armadillos or humans, and thus zoonotic transmission of *M*. *leprae*, may be possible. However, *M*. *leprae* DNA was not found in the marmosets examined in this study, so such pathogen transmission likely did not occur in this population.

Conversely, out of the 98 marmosets tested, 14 were positive for some mycobacterial DNA, specifically the rpoB1 locus (Fig 3). The rpoB1 primer/probe set can amplify species within and outside the MTBC [17], so positive amplification of this target only suggests that DNA from some mycobacterial species related to the MTBC is present. Several of the rpoB1 positive marmosets show late qPCR amplification signals, which reflect results produced by mismatches between the primer/probe and endogenous DNA and suggest that the DNA present in these marmosets likely belongs to a mycobacterial species distantly related to the MTBC. Although this study did not identify any IS6110 positives, previous studies have identified the presence of this locus in New World primates [37]. However, the IS6110 mobile element has been found to be homologous in some related soil mycobacteria [5], and targeting this element with PCR has previously produced false-positives [38]. Therefore, the lack of IS6110 positives in this study rules out this line of potential false positives.

Targeted capture and sequencing of mycobacterial genes in these rpoB1 positive marmosets reveal that the rpoB1 locus detected via qPCR is not from a member of the MTBC, as sample reads did not align to any MTBC reference genome. Rather, metagenomic analyses indicate that the detected rpoB1 locus may come from a different pathogenic or non-pathogenic species in the *Mycobacterium* genus (Table 3). These species include the pathogenic *M*. *avium* which is known to infect birds, humans, and some other animals, the pathogenic *M*. *leprae* which is known to infect armadillos, humans, and some other animals, and the non-pathogenic *M*. *gilvum*, *M*. *chubuense*, *M*. *neoaurum*, *M*. *smegmatis*, *M*. *vanbaalenii*, *M*. *sp*.*FI-13364*, *M*. *sp*. *JDM601*, *and M*. *sp*. *VKM Ac-1817D*. *M*. *leprae is not a likely candidate given the results of the 85B and rlep performed in this study*. *M*. *avium* has the most assigned reads which is interesting since it is the species in this list that is most closely related to *M*. *tuberculosis*. However, since relatively few reads were obtained from this sequencing technique (Table 2), this study cannot conclusively identify the specific mycobacterial species infecting rpoB1 positive marmosets. Nevertheless, these results do confirm the presence of mycobacterial DNA, which in turn confirms the validity of qPCR results from the described method.

Marmosets testing positive for mycobacterial DNA include both species (*C*. *jacchus* and *C*. *penicillata*) and their hybrids (Fig 4). Although the presence of DNA does not equate the presence of viable cells, these finding do suggest that these marmosets likely harbored mycobacterial cells. Additionally, this further implies that all examined taxa may be capable of acquiring and harboring mycobacteria. Although marmosets are susceptible to several human infectious diseases [20], this is the first study to identify potentially zoonotic mycobacterial DNA in wild marmoset populations. All sampled marmosets were found near urbanized areas, and although cases of infection were not found in all locations, several marmoset populations were found to harbor mycobacterial DNA. These potential infections appear to be concentrated in and around the neighboring cities of Silva Jardim and Rio Bonito in the state of Rio de Janeiro, the neighboring municipalities of Petrolina, Pernambuco and Juzaeiro, Bahia, and in Recife, Pernambuco. Marmosets in these areas were documented as experiencing high frequencies of direct and indirect human contact, such as time spent at a rehabilitation center, direct human provisioning, close proximity to major highways, and close proximity to domesticated animals. In particular, Rio de Janeiro state is an area that has grown tremendously in the last 20 years due to an off shore oil business (personal observation, J. Malukiewicz). It has experienced heavy deforestation, highway expansions, and growing human populations. Marmosets were recently introduced into this urban area, and because they are not native to this developing region, they may be exposed to increased human contact. This in turn may make marmosets in these areas more susceptible to mycobacterial infection.

Overall, these findings reveal that wild marmoset populations, especially those in close contact with human populations, may harbor mycobacterial cells which may lead to downstream infections. This also suggests that these primates may serve as zoonotic reservoirs for mycobacterial pathogens. Close contact between humans and other animals is known to increase the spread of zoonotic diseases [1,13]. As evidenced by this study and others, non-human primates can harbor mycobacterial DNA, which suggests that these animals may be susceptible to mycobacteria, such as MTBC and *M*. *leprae* [5,8,12]. Therefore, as humans continue expanding into natural animal habits, it is important to assess the distribution of primates serving as zoonotic pathogen reservoirs. Further, this study validates that qPCR techniques can be used to accurately survey for the presence of mycobacterial DNA among wild primate populations. PCR is categorized as a useful clinical tool for identifying mycobacterial DNA in humans [39], and this is especially true in developing countries with difficult field conditions [40] and when individual infection levels are subclinical but high enough for pathogens to be transmitted to unaffected patients [35]. In humans, PCR techniques have primarily utilized blood, skin biopsy, nasal swab, and saliva samples [32–34,40], but none of these tissues is ideal in regards to reduced invasiveness and optimized testing accuracy. The present study identifies a technique that combines five qPCR assays and utilizes cheek swab samples. Although this specific method has only been tested in non-human animals, it may be useful for detecting mycobacterial DNA in humans, as well.

In conclusion, marmosets living in close proximity to humans in north- and south-eastern Brazil can harbor potentially zoonotic mycobacteria in natural settings. TaqMan qPCR assays serve as accurate techniques for the detection of DNA from such mycobacteria and should be used, possibly in combination with sequencing techniques, to identify pathogen exposed primate populations. This is crucial as such populations can serve as reservoirs and amplify zoonotic disease transmissions between human and non-human primates.

## Supporting Information

S1 Text Supplementary Tables A-C. (DOCX)

S1 Dataset Sequence read data. (ZIP)
