# Correction: Validation of qPCR Methods for the Detection of *Mycobacterium* in New World Animal Reservoirs

**DOI:** 10.1371/journal.pntd.0004756

**Published:** 2016-05-24

**Authors:** Genevieve Housman, Joanna Malukiewicz, Vanner Boere, Adriana D. Grativol, Luiz Cezar M. Pereira, Ita de Oliveira e Silva, Carlos R. Ruiz-Miranda, Richard Truman, Anne C. Stone

In the fourth paragraph of the Discussion section, reference #37 and #5 are switched. The paragraph should read as follows: "Although this study did not identify any IS6110 positives, previous studies have identified the presence of this locus in New World primates [5]. However, the IS6110 mobile element has been found to be homologous in some related soil mycobacteria [37], and targeting this element with PCR has previously produced false-positives [38]."
