# Supplementary material for: Validation of qPCR Methods for the Detection of Mycobacterium in New World Animal Reservoirs
Source: PLoS Negl Trop Dis. 2015 Nov 16;9(11):e0004198. doi: 10.1371/journal.pntd.0004198 (PMC4646627; doi:10.1371/journal.pntd.0004198)
Supplement: S1 Text — (DOCX) [file pntd.0004198.s001.docx]

**S1 Text. Supplementary Tables A-C.**

**Table A. qPCR Primer and Probe Sequences.**

| **Primer/Probe** | **Sequence (5' 🡪 3')** |
| --- | --- |
| rpoB1 Forward Primer | GGGTTGTTCTGGTCCATGAATT |
| rpoB1 Probe | CTCAGCTGGCTGGTG |
| rpoB1 Reverse Primer | GGTCGCCGCGATCAAG |
| rpoB2 Forward Primer | CAACGTCGAGGTGCTATCG |
| rpoB2 Probe | TCGCCGCACCGTCACT |
| rpoB2 Reverse Primer | CTCCAGGTCCTCGTCCTCA |
| IS6110 Forward Primer | GGGTAGCAGACCTCACCTATGTG |
| IS6110 Probe | ACCTGGGCAGGGTT |
| IS6110 Reverse Primer | CGGTGACAAAGGCCACGTA |
| 85B Forward Primer | GTGGTCGGCCTCTCGAT |
| 85B Probe | CTCGGCCCTAATACT |
| 85B Reverse Primer | CGAGCCAGCATAGATGAACTGATC |
| rlep Forward Primer | GCAGTATCGTGTTAGTGAA |
| rlep Probe | TCGATGATCCGGCCGTCGGCG |
| rlep Reverse Primer | CGCTAGAAGGTTGCCGTATG |

Primer and probe sequences used for the TaqMan® qPCR assays. These assays included two targeting the single-copy rpoB gene (rpoB1 and rpoB2 [17]), one targeting the MTBC-specific multi-copy IS6110 insertion element [4,18], one targeting the 85B single-copy M. leprae gene [16], and one targeting the rlep multi-copy M. leprae gene [15].

**Table B. qPCR Primer and Probe Specificity using BLAST.**

|  | **rpoB1** | | | **rpoB2** | | | **IS6110** | | | **85B** | | | **rlep** | | |
| --- | --- | --- | --- | --- | --- | --- | --- | --- | --- | --- | --- | --- | --- | --- | --- |
|  | **Forward** | **Probe** | **Reverse** | **Forward** | **Probe** | **Reverse** | **Forward** | **Probe** | **Reverse** | **Forward** | **Probe** | **Reverse** | **Forward** | **Probe** | **Reverse** |
| Bacteria (total hits) | 264 hits | 376 hits | 200 hits | 910 hits | 734 hits | 442 hits | 200 hits | 808 hits | 195 hits | 832 hits | 268 hits | 274 hits | 400 hits | 200 hits | 390 hits |
| Actinobacteria | 97.35% | 88.03% | 90.50% | 100.00% | 98.23% | 100.00% | 100.00% | 97.40% | 100.00% | 19.35% | 2.61% | 56.93% | 77.75% | 87.50% | 80.26% |
| Mycobacterium | 97.35% | 84.31% | 87.00% | 100.00% | 97.14% | 86.65% | 100.00% | 96.78% | 100.00% | 5.41% | 2.24% | 56.20% | 77.75% | 24.00% | 78.97% |
| Mycobacterium tuberculosis complex | 97.35% | 81.12% | 86.00% | 100.00% | 96.46% | 86.65% | 100.00% | 96.78% | 100.00% | - | - | - | - | - | - |
| Mycobacterium tuberculosis | 94.70% | 77.13% | 84.00% | 92.75% | 88.83% | 81.45% | 97.50% | 96.16% | 100.00% | - | - | - | - | - | - |
| Mycobacterium bovis | 2.65% | 3.99% | 2.00% | 4.18% | 4.22% | 2.71% | 2.50% | 0.62% | 2.56% | - | - | - | - | - | - |
| Mycobacterium canettii | - | - | - | 3.08% | 3.41% | 2.49% | - | - | - | - | - | - | - | - | - |
| Mycobacterium kansasii | - | - | 1.00% | - | - | - | - | - | - | - | - | - | - | - | - |
| Mycobacterium intracellulare | - | 1.60% | - | - | - | - | - | - | - | - | - | - | - | - | - |
| Mycobacterium haemophilum DSM | - | 1.60% | - | - | 0.68% | - | - | - | - | 0.36% | - | - | - | - | - |
| Mycobacterium sp. EPa45 | - | - | - | - | - | - | - | - | - | 0.36% | - | 0.36% | - | 0.50% | - |
| Mycobacterium leprae | - | - | - | - | - | - | - | - | - | 0.84% | 2.24% | 55.84% | 77.75% | 21.00% | 78.97% |
| Mycobacterium smegmatis | - | - | - | - | - | - | - | - | - | 0.60% | - | - | - | - | - |
| Mycobacterium avium complex | - | - | - | - | - | - | - | - | - | 0.48% | - | - | - | - | - |
| Mycobacterium abscessus | - | - | - | - | - | - | - | - | - | 2.16% | - | - | - | - | - |
| Mycobacterium rhodesiae NBB3 | - | - | - | - | - | - | - | - | - | 0.60% | - | - | - | 0.50% | - |
| Mycobacterium neoaurum VKM | - | - | - | - | - | - | - | - | - | - | - | - | - | 0.50% | - |
| Mycobacterium gilvum | - | - | - | - | - | - | - | - | - | - | - | - | - | 1.00% | - |
| Mycobacterium vanbaalenii PYR-1 | - | - | - | - | - | - | - | - | - | - | - | - | - | 0.50% | - |
| Other Actinobacteria | - | 3.72% | 3.50% | - | 1.09% | 13.35% | - | 0.62% | - | 13.94% | 0.37% | 0.73% | - | 63.50% | 1.28% |
| Proteobacteria | 2.65% | 11.97% | 9.00% | - | 1.77% | - | - | 1.73% | - | 76.68% | 90.67% | 26.28% | 14.75% | 10.00% | 13.33% |
| Other Bacteria | - | - | 0.50% | - | - | - | - | 0.87% | - | 3.97% | 6.72% | 16.79% | 7.25% | 2.50% | 6.41% |

BLAST was used to compare each TaqMan® qPCR primer and probe sequence against the Nucleotide collection (nr/nt) using blastn (optimized for somewhat similar sequences). All other settings were default, and only matches (hits) against Bacteria were considered. The total number of Bacterial hits are listed as well as the percentage of these hits that came from different bacterial taxa. As detailed in the table, rpoB1 is specific to *Mycobacterium*, rpoB2 and IS6110 are specific to the MTBC, and 85B and rlep are mostly specific to *M. leprae*. However, sequences from non-mycobacterial taxa do account for some hits.

**Table C. qPCR Inhibition Test Results.**

| **qPCR Target** | **Sample** | | **Mean Ct Value** | **Ct Standard Error** | **Significance** |
| --- | --- | --- | --- | --- | --- |
| rpoB1 | Control | | 23.052 | 0.171 |  |
|  | RJ015 | positive marmoset | 23.270 | 0.264 | not significant |
|  | CJA018 | positive marmoset | 23.571 | 0.371 | not significant |
|  | RJ007 | negative marmoset | 23.441 | 0.267 | not significant |
|  | RJ009 | negative marmoset | 23.463 | 0.144 | not significant |
| rpoB2 | Control | | 23.284 | 0.208 |  |
|  | RJ021 | negative marmoset | 23.416 | 0.095 | not significant |
|  | PJ046 Ita | negative marmoset | 23.533 | 0.126 | not significant |
| IS6110 | Control | | 19.610 | 0.042 |  |
|  | CPE016 | negative marmoset | 21.351 | 0.079 | p < 0.01 |
|  | PJ031 | negative marmoset | 19.845 | 0.038 | not significant |
| 85B | Control | | 23.177 | 0.033 |  |
|  | CPE024 | negative marmoset | 23.822 | 0.105 | p < 0.01 |
|  | CJA021 | negative marmoset | 23.468 | 0.008 | not significant |
|  | Dno021 | positive armadillo | 23.608 | 0.158 | not significant |
|  | Dno042 | positive armadillo | 23.626 | 0.051 | p < 0.01 |
|  | Dno003 | false negative armadillo | 23.687 | 0.124 | not significant |
|  | Dno008 | false negative armadillo | 23.551 | 0.140 | not significant |
|  | Dno030 | true negative armadillo | 23.453 | 0.063 | p < 0.01 |
|  | Dno045 | true negative armadillo | 23.783 | 0.227 | not significant |
| Rlep | Control | | 16.839 | 0.074 |  |
|  | PJ056 | negative marmoset | 16.900 | 0.107 | not significant |
|  | CJA040 | negative marmoset | 17.159 | 0.026 | not significant |
|  | Dno024 | positive armadillo | 17.250 | 0.119 | not significant |
|  | Dno040 | positive armadillo | 17.305 | 0.206 | not significant |
|  | Dno005 | false negative armadillo | 17.131 | 0.094 | not significant |
|  | Dno017 | false negative armadillo | 17.171 | 0.032 | not significant |
|  | Dno029 | true negative armadillo | 17.574 | 0.458 | not significant |
|  | Dno038 | true negative armadillo | 17.302 | 0.153 | not significant |

For each targeted locus, qPCR inhibition tests were performed using subsets of relevant samples. These include marmosets that were identified as positive during the study (positive marmosets), marmosets that were identified as negative (negative marmosets), armadillos that were identified as positive (positive armadillos), armadillos that were experimentally infected with *M. leprae* but then falsely identified as negative (false negative armadillo), and non-infected armadillos that were correctly identified as negative (true negative armadillo). Additionally, marmoset samples represented all taxa and locations. Each qPCR assay was set up as described in the methods. However, each sample had 6 replicates – 3 replicates contained only sample DNA extract, and 3 replicates contained sample DNA extract that was spiked with 1uL of a positive control. Samples amplified as expected, and the mean Ct values of spiked samples were statistically compared to that of the control sample using t-tests. The Ct values of most spiked samples did not significantly differ from those of the controls, so overall, there does not appear to be any major inhibition effects across samples.
