# Supplementary material for: Validation of qPCR Methods for the Detection of Mycobacterium in New World Animal Reservoirs
Source: PLoS Negl Trop Dis. 2015 Nov 16;9(11):e0004198. doi: 10.1371/journal.pntd.0004198 (PMC4646627; doi:10.1371/journal.pntd.0004198)
Supplement: S1 Dataset — (ZIP) [file pntd.0004198.s002.zip › S1 Dataset Description.docx]

**S1 Dataset. Sequence Read Data.**

**Description of Files.**

Zipped folder contains marmoset mycobacterial DNA sequence reads for different samples (n=8) and one blank. Samples include CJA024, CJA025, CPE045, CPE046, RJ025, RJ026, RJ031, and RJ036, and the blank is LB_GH.

DNA libraries were prepared according to [26-27] and enriched as in [28] for MTBC DNA using baits designed to capture the MTBC rpoB, katG, gyrA, gyrB, and mtp40 genes and sequenced on an Illumina MiSeq Nano. Reads provided here include merged forward and reverse reads with artifacts removed [27]. Each sample and blank has one file containing all sequence reads. These files include:

Sample_CJA024.FandR_merged.txt.

Sample_CJA025.FandR_merged.txt.

Sample_CPE045.FandR_merged.txt.

Sample_CPE046.FandR_merged.txt.

Sample_RJ025.FandR_merged.txt.

Sample_RJ026.FandR_merged.txt.

Sample_RJ031.FandR_merged.txt.

Sample_RJ036.FandR_merged.txt.

Sample_LB_GH.FandR_merged.txt.

As described in the paper, quality filtering [27], BWA alignment [29], and MEGAN analyses [30] were performed using these sequence reads. These files are not provided here but are available upon request.
